# Autocrine signaling is a key regulatory element during osteoclastogenesis

**DOI:** 10.1242/bio.20148128

**Published:** 2014-07-25

**Authors:** Paul Kopesky, Kerstin Tiedemann, Dahlia Alkekhia, Christoph Zechner, Bjorn Millard, Birgit Schoeberl, Svetlana V. Komarova

**Affiliations:** 1 Merrimack Pharmaceuticals, One Kendall Square, Suite B7201, Cambridge, MA 02139, USA; 2 Shriners Hospital for Children – Canada, 1529 Cedar Avenue, Montreal, QC H3G IA6, Canada; 3 Faculty of Dentistry, McGill University, 3640 rue University, Montreal, QC H3A 0C7, Canada

**Keywords:** Cytokine, High content imaging, Interleukin-8, Osteoclastogenesis, PLSR

## Abstract

Osteoclasts are responsible for bone destruction in degenerative, inflammatory and metastatic bone disorders. Although osteoclastogenesis has been well-characterized in mouse models, many questions remain regarding the regulation of osteoclast formation in human diseases. We examined the regulation of human precursors induced to differentiate and fuse into multinucleated osteoclasts by receptor activator of nuclear factor kappa-B ligand (RANKL). High-content single cell microscopy enabled the time-resolved quantification of both the population of monocytic precursors and the emerging osteoclasts. We observed that prior to induction of osteoclast fusion, RANKL stimulated precursor proliferation, acting in part through an autocrine mediator. Cytokines secreted during osteoclastogenesis were resolved using multiplexed quantification combined with a Partial Least Squares Regression model to identify the relative importance of specific cytokines for the osteoclastogenesis outcome. Interleukin 8 (IL-8) was identified as one of RANKL-induced cytokines and validated for its role in osteoclast formation using inhibitors of the IL-8 cognate receptors CXCR1 and CXCR2 or an IL-8 blocking antibody. These insights demonstrate that autocrine signaling induced by RANKL represents a key regulatory component of human osteoclastogenesis.

## INTRODUCTION

Osteoclasts are responsible for the physiological resorption of calcified tissues during skeletal development and bone remodeling, and for bone destruction in osteoporosis, periodontitis and cancer metastases to bone (Burr, 2002; Honore et al., 2000; Kong et al., 1999; Pearse et al., 2001; Ritchlin et al., 2003; Teng et al., 2000). Osteoclasts are terminally differentiated multinucleated cells formed by fusion from monocytic precursors. To resorb bone, osteoclasts attach to the bone matrix, lower the extracellular pH to dissolve hydroxyapatite, and secrete proteolytic enzymes, cathepsin K and matrix metalloproteinase-9 (MMP-9), to digest the organic matrix (Blair et al., 2011). Specific osteoclast marker genes include tartrate-resistant acid phosphatase (TRAP), integrin α_v_β_3_ and calcitonin receptor (Boyle et al., 2003; Henriksen et al., 2011).

Two cytokines, receptor activator of nuclear factor κB ligand (RANKL) and macrophage colony stimulating factor (MSCF), were identified as the key regulators of osteoclast formation and function. RANKL, expressed by cells of osteoblastic origin, lymphocytes and cancer cells (Lacey et al., 1998; Manabe et al., 2001; Yasuda et al., 1998) acts through its receptor RANK, expressed on osteoclast precursors and mature cells (Wada et al., 2006). Signaling through RANK leads to activation of the transcription factors NF-κB and AP-1 and the mitogen-activated protein kinases JNK, ERK and p38 (Boyle et al., 2003). In addition, expression and activation of the transcription factor nuclear factor of activated T cells (NFAT) c1 is essential for osteoclastogenesis (Ishida et al., 2002; Takayanagi et al., 2002). Inactive hyper-phosphorylated NFATc1 is maintained in the cytosol. Calcium signaling, in particular calcium oscillations observed in osteoclast precursors, stimulate phosphatase calcineurin, which dephosphorylates NFATc1 allowing its nuclear translocation (Hwang and Putney, 2011; Takayanagi et al., 2002). RANKL in combination with macrophage colony-stimulating factor (MCSF) was shown to be necessary and sufficient to induce osteoclast formation and bone resorption, and became an attractive therapeutic target. Denosumab, a monoclonal antibody targeting RANKL is now used clinically to treat post-menopausal osteoporosis and prevent skeletal-related events in patients with bone metastasis (Smith et al., 2013). Nevertheless, many aspects of osteoclastogenesis remain unclear and accelerated remodeling, especially associated with inflammatory conditions, such as rheumatoid arthritis and periodontitis, remains poorly tractable.

In this study, we used human osteoclast precursors and a Systems Biology approach combining high-content imaging with quantitative cytokine profiling and statistical modeling to identify downstream regulators of RANKL-induced osteoclastogenesis. High-content imaging enabled the time resolved quantification of the population of monocytic osteoclast precursors as well as formation of mature osteoclasts as a function of RANKL and inhibitor dose. By correlating comprehensive profiles of osteoclastogenesis phenotype with secreted cytokine levels, we identified IL-8 as a key RANKL-induced autocrine regulator of NFATc1 activation and osteoclast formation.

## RESULTS

### Characterizing coordinated osteoclastogenesis *in vitro*

**Fig. 1.**
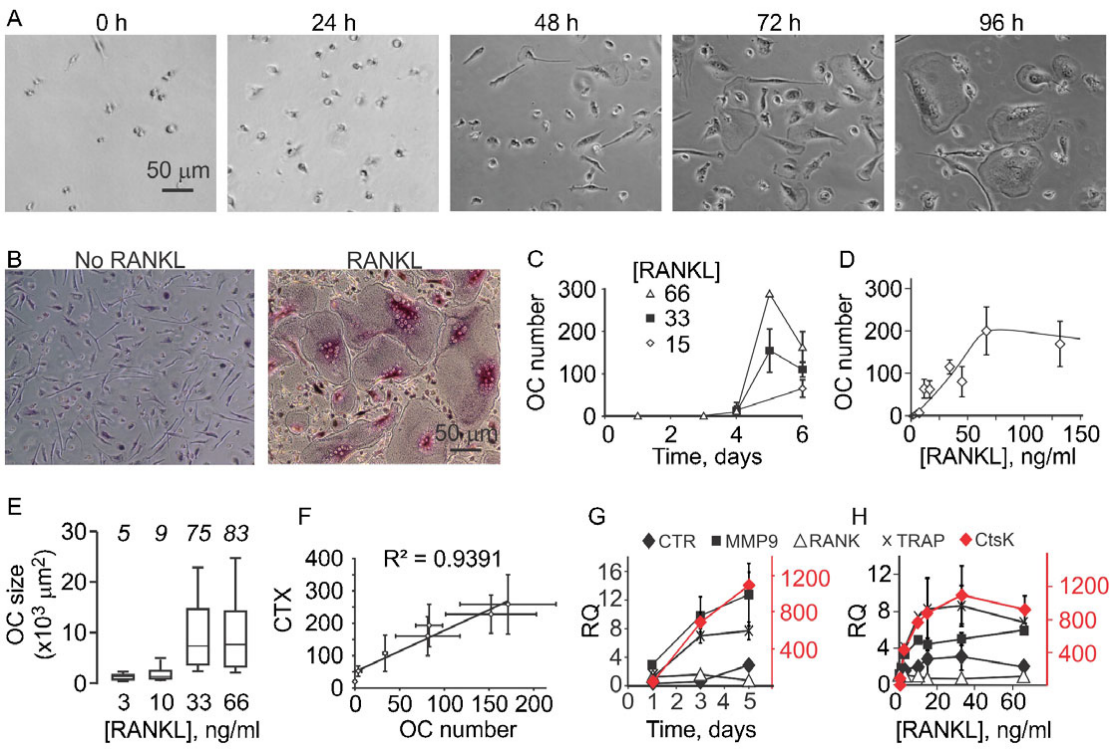
Dynamics of osteoclastogenesis. (A,B) Human osteoclast precursors where treated with MCSF (33 ng/ml) and RANKL (33 ng/ml) and representative images were obtained. (A) Live osteoclast differentiation cultures at different time after plating. (B) Fixed, TRAP stained cells cultured for 5 days without (left) or with RANKL (right). Scale bars: 50 µm (applies to all images). (C) Human precursors were cultured with MCSF (33 ng/ml) and RANKL (15, 33 and 66 ng/ml) for 0–6 days, and osteoclasts were manually counted. Data are means ± SEM, *n* = 3 independent experiments. (D,E) Human precursors were cultured with MCSF (33 ng/ml) and RANKL (0–132 ng/ml) for 5–6 days, and osteoclasts were manually counted (D) or osteoclast planar area was measured (E). For panel D, data are means ± SEM, *n* = 2–10 independent experiments. For panel E, the box-plots indicate the minimal and maximal values (whiskers), the 25th and 75th percentiles (the limits of the box), and the median values (the line within the box), *n* for each concentration is shown in italics. (F) Human precursors were cultured on osteoassay bone plate with MCSF (33 ng/ml) and RANKL (0–264 ng/ml) for 6 days, then the osteoclast numbers were counted (plotted on the *x*-axis) and the amount of degraded collagen peptides (CTX) in conditioned medium was measured (plotted on the *y*-axis). Data are means ± SEM, *n* = 2 independent experiments. (G,H) Human precursors were cultured with MCSF (33 ng/ml) and RANKL (0–66 ng/ml) for 1–6 days and the expression of cathepsin K (CtsK, scale on the right), calcitonin receptor (CTR), MMP-9, RANK and TRAP was assessed, normalized to endogenous control GAPDH and expressed relative to samples cultured without RANKL. Data are means ± SEM, *n* = 2–4 independent experiments. (G) Time course of changes in gene expression of osteoclast marker genes in cultures treated with 33–66 ng/ml RANKL. (H) RANKL concentration dependence of changes in gene expression of osteoclast marker genes on day 5–6.

Human osteoclast precursors successfully differentiated into mature osteoclasts after addition of RANKL (33 ng/ml) and MCSF (33 ng/ml) (Fig. 1A). While no osteoclasts were formed when the cultures were treated with MCSF only (Fig. 1B), when RANKL and MCSF were added we observed that large, multinucleated osteoclasts that stain positive for TRAP (Fig. 1B) emerged after 4 days of culture (Fig. 1C). Osteoclasts were formed in the numbers proportional to added RANKL in the concentration range between 0–66 ng/ml. Higher concentrations of RANKL did not provide additional stimulation of osteoclast formation (Fig. 1D), suggesting the saturation of osteoclastogenic responses at higher that 66 ng/ml RANKL. Alternatively, it is possible that the size of the culture well, rather than inability to further differentiate limited osteoclast formation at higher levels of RANKL. Only small osteoclasts were formed at low concentration of RANKL, while at concentrations of 33 ng/ml and higher an average osteoclast size increased 5-fold (Fig. 1E). Osteoclasts formed *in vitro* were functional as evident by their resorptive ability (Fig. 1F). We examined the time course (Fig. 1G) and RANKL concentration dependence (Fig. 1H) for the expression of osteoclast marker genes, including differentiation markers, RANK, TRAP, and calcitonin receptor (CTR), and functionality markers cathepsin K and MMP-9. The expression of osteoclastogenic genes was not affected by changes in MCSF (data not shown). In the presence of RANKL, the expression of TRAP, MMP9 and cathepsin K was strongly up-regulated at day 3 after induction of differentiation, prior to active osteoclast fusion (Fig. 1G), and the expression of CTR increased at day 5. The expression of all osteoclast markers, except RANK, exhibited strong RANKL concentration dependence with the half maximum at [RANKL] of 3–10 ng/ml, considerably lower than [RANKL] required for full osteoclastogenesis.

### Analysis of different cell populations using high content imaging

**Fig. 2.**
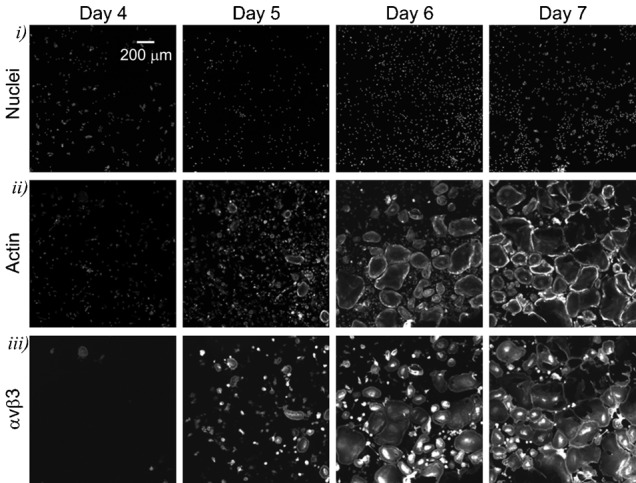
Studying osteoclastogenesis using high-content imaging. Human osteoclast precursors were cultured with MCSF (33 ng/ml) and RANKL (33 ng/ml) for 4–7 days, fixed and stained with Hoechst for nuclei, phalloidin for actin as a cytoplasmic marker, and for α_v_β_3_ integrin as an osteoclast marker. Representative images demonstrate temporal changes in the appearance of nuclei (top) and actin (middle), and emergence of α_v_β_3_ integrin (bottom) during osteoclast differentiation. Scale bar: 200 µm (applies to all images).

**Fig. 3.**
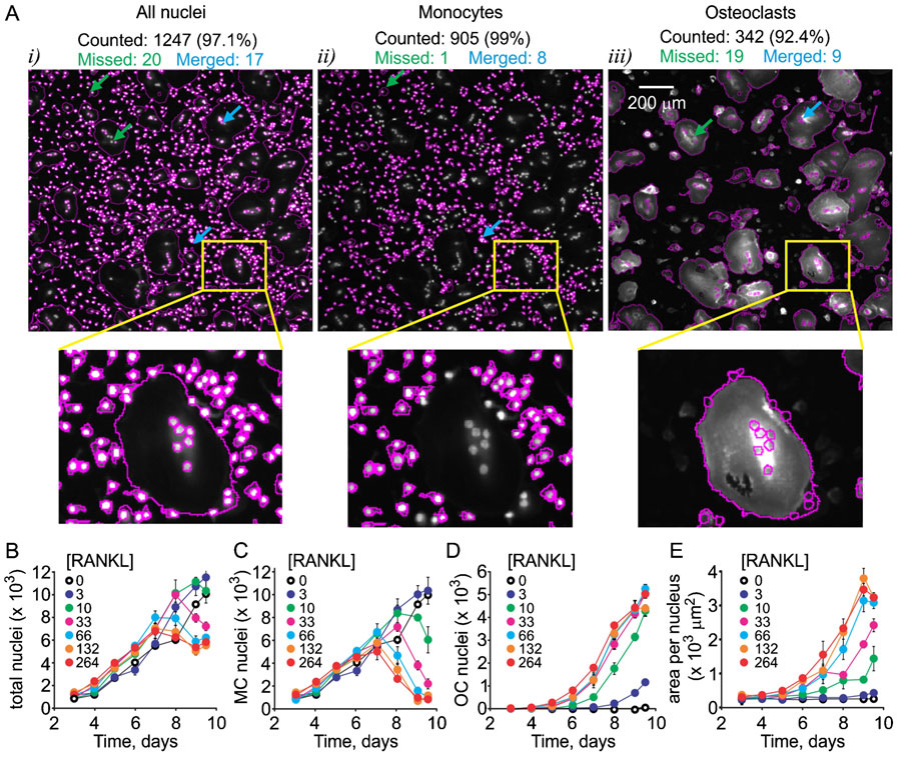
Quantification of cellular dynamics using high content imaging. Human osteoclast precursors were cultured with MCSF (33 ng/ml) and RANKL (0–264 ng/ml) for 3–9.5 days, fixed and stained for nuclei/cytoplasm (Whole Cell Blue)/α_v_β_3_. (A) The quantification of total number of nuclei (*i*), monocyte nuclei (*ii*) and osteoclast nuclei (*iii*) was performed using a custom osteoclast quantification module for the open source software package ImageRail and validated manually. Scale bar is 200 µm. Total numbers counted are given in black, green arrows and numbers are nuclei missed by automatic quantification, blue arrows and numbers are merged nuclei miscounted by automatic quantification. The smaller images below are magnifications of a region indicated by a yellow triangle. (B–E) Time and [RANKL] dependences of changes in the total numbers of nuclei (B), numbers of monocyte nuclei (C), numbers of osteoclast nuclei (D) and cytoplasm area per nucleus (E) were obtained using high content imaging and automatic quantification. Data are means ± SEM, *n* = 6 replicates, representative data from 1 of 4 independent experiments are shown. Two-way ANOVA indicates significant (p<0.0001) effect of time and RANKL concentration as well as significant interaction between these factors for all 4 experimental outcomes.

Osteoclastogenesis *in vitro* occurs in a heterogeneous system containing at least two cell types – monocytes and osteoclasts – and is known to be characterized by complex cell–cell interactions (Akchurin et al., 2008). We examined the changes in both cell populations with time using a high content imaging (Fig. 2). The cultures in 96-well plates were fixed at different time after induction of osteoclastogenesis and stained for nucleic acids using Hoechst, for actin (which in monocytes and inactive osteoclasts forms diffuse cytoplasmic stain) using phalloidin, and for osteoclast marker integrin α_v_β_3_ (CD51/CD61) using immunofluorescence. We observed a time-dependent increase in the total nuclei numbers (Fig. 2*i*) and cell size of an osteoclastic subpopulation (Fig. 2*ii*), evident by emergence of osteoclast differentiation marker α_v_β_3_ (Fig. 2*iii*). Automating the quantification of osteoclast numbers is challenging due to exceptional diversity in size and shape of these cells. Separation of monocyte and osteoclast nuclei represents an additional computational problem. Semi-automatic imaging solution ImageRail (http://www.semanticbiology.com/software/imagerail), previously developed for high content microscopy (Millard et al., 2011) was successfully adapted for quantitative analysis of osteoclast cultures (Fig. 3). All the nuclei in the image (Fig. 3A*i*) were segregated as either osteoclastic if they resided in α_v_β_3_ positive cells with ≧2 nuclei (Fig. 3A*iii*) or monocytic for all other mononuclear cells (Fig. 3A*ii*). We have manually validated the nuclear counts, which was 91–99% accurate in all instances, with inaccuracies attributed to missed nuclei (0.1–5%) or overlapping (merged) nuclei (1–2%). This technique allowed for unbiased, time-resolved quantification of each cell type as a function of RANKL concentration during osteoclastogenesis (Fig. 3B–E). Total numbers of nuclei continuously increased over time when cells were cultured without RANKL, or at low concentrations of RANKL (3–10 ng/ml). At high RANKL concentrations (33–264 ng/ml) the nuclei numbers increased similarly during the first 7–8 days of culture, after which they reached a plateau (Fig. 3B). The monocyte and osteoclast nuclei changed in a complementary RANKL-dependent manner – in the cultures exposed to higher concentrations of RANKL, early drop in the numbers of monocyte nuclei (Fig. 3C) corresponded to the time of increase in osteoclast nuclei (Fig. 3D), confirming that the main cause of the drop in monocyte nuclei was the fusion of monocytes into the osteoclasts. Over a 10 day culture period no nuclear fragmentation or condensation was detected (data not shown). We observed significant RANKL- and time-dependent increase in osteoclast size (Fig. 3E), which likely explains the lower total numbers of nuclei in late cultures treated with high concentrations of RANKL. One important result of these experiments is that at low levels of RANKL the osteoclastogenesis was delayed rather than prevented, so that even after exposure to as low as 3 ng/ml RANKL, osteoclasts started to appear on day 9 of the culture. It is important to stress that these data represent the simultaneous analysis of 4 single cell attributes examined in 10^3^–10^4^ cells per condition in 7 experimental conditions performed at least in triplicate at 8 different time points, representing 10^5^–10^7^ individual entries.

### Intracellular signaling events important for osteoclastogenesis

**Fig. 4.**
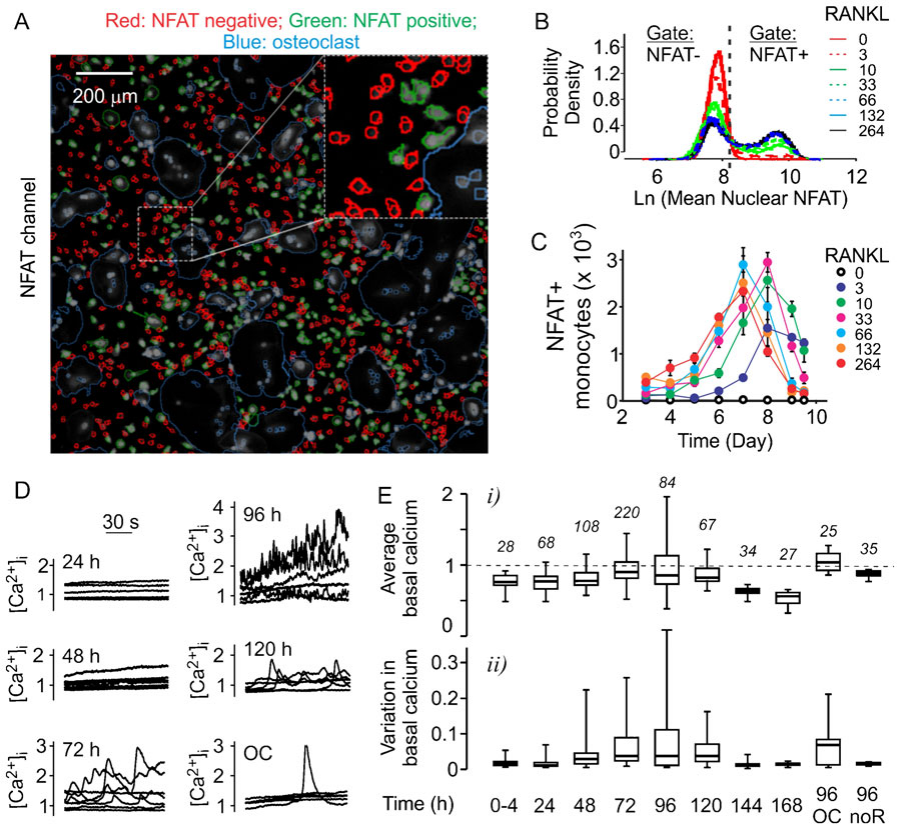
Dynamic changes in calcium/NFATc1 pathway during osteoclastogenesis. (A–C) Human precursors were treated with MCSF (33 ng/ml) and RANKL (0–264 ng/ml) for 0–9.5 days, fixed, stained for nuclei/cytoplasm (Whole Cell Blue)/α_v_β_3_ for automated classification of monocytic and osteoclastic cells and for NFATc1. (A) Representative images demonstrating the separation of NFATc1-negative (red) and NFATc1-positive (green) nuclei. Blue outlines represents osteoclasts identified using α_v_β_3_ channel. Scale bar: 200 µm. (B) Representative probability density function of mean nuclear NFATc1 signal in monocytes on day 7. Dashed vertical line represents computationally estimated threshold between the NFATc1-negative and positive nuclei. (C) Time- and [RANKL]-dependence of changes in the numbers of monocytes with nuclear NFATc1. Data are means ± SEM, *n* = 6 replicates from 1 of 3 independent experiments. (D,E) Changes in calcium signaling during osteoclastogenesis. Samples of osteoclast precursors cultured with MCSF (33 ng/ml) and RANKL (33 ng/ml) were loaded daily with fura-2AM and changes in [Ca^2+^]_i_ were monitored for 120 s. (D) Representative traces demonstrate basal [Ca^2+^]_i_ in 5–8 individual monocytes per culture time or in individual osteoclasts (OC) at 96 h. 30 s bar indicating the time scale applies to all traces. (E) Box plots of the average [Ca^2+^]_i_ (*i*) and variation in basal [Ca^2+^]_i_ (*ii*) in monocytes treated with RANKL for indicated time, osteoclasts formed after 96 h of RANKL treatment (96 OC) and monocytes cultured without RANKL for 96 h (96 noR). The number of cells per condition is indicated in italics.

The additional fluorescent imaging channel is available to study the changes in other proteins of interest, such as an osteoclastogenic transcription factor NFATc1, which translocates to the nuclei upon activation (Fig. 4A). Using high content imaging, we have examined the dynamics of NFATc1 activation during osteoclastogenesis (Fig. 4A–C). We have found that differentiation of osteoclasts coincides with dramatic increase in the numbers of NFATc1-positive monocytic cells (Fig. 4C). The maximum level of NFATc1 activation was achieved earlier when cells were exposed to higher concentrations of RANKL. While the total numbers of NFATc1-positive monocytes decreased after day 7, this reflects a decrease in the total number of monocytes, since the proportion of NFATc1-positive monocytes remained within 30–45% between day 6 and 8. Since NFATc1 nuclear translocation is induced by elevations of cytosolic free calcium ([Ca^2+^]_i_), we next examined how calcium levels change during osteoclastogenesis. We observed subtle change in basal Ca^2+^ levels within 24 h after addition of RANKL, which developed into persistent high magnitude fluctuations in the basal Ca^2+^ by day 3–4 of culture. By day 5–6 the fluctuations diminished, resulting in stabilization of basal calcium in osteoclasts (Fig. 4D, *OC*). Both the average basal Ca^2+^ levels (Fig. 4E*i*), and variations in basal Ca^2+^ levels calculated for each cell as a standard deviation of basal [Ca^2+^]_i_ over 120 s of measurement (Fig. 4E*ii*) progressively increased during osteoclastogenesis till day 4, when fusion of mononucleated cells is generally observed. After 5 days of RANKL treatment, calcium levels and variation in monocytes declined (Fig. 4E, *120–144 h*). In osteoclasts, basal Ca^2+^ levels remained increased, while active fluctuations subsided. Importantly, human precursors cultured for 5 days without RANKL did not demonstrate significant changes in the level or variation in basal [Ca^2+^]_i_ (Fig. 4E, *noR*). These data indicate that calcium/NFATc1 pathway is strongly induced at the fusion stage of osteoclastogenesis.

**Fig. 5.**
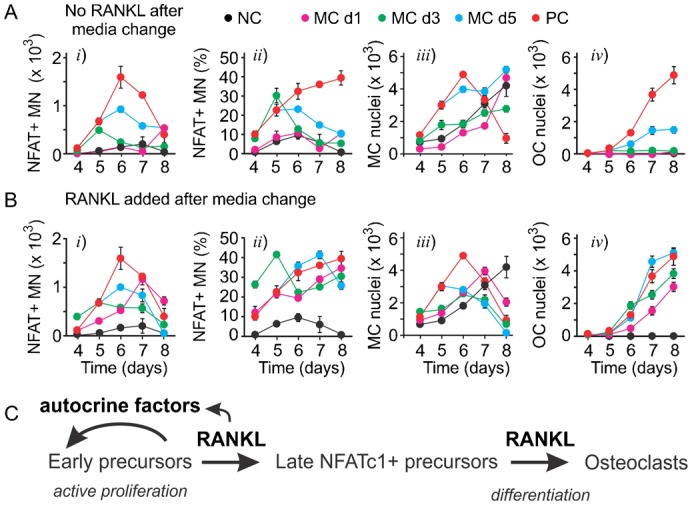
RANKL induces production of autocrine factors that regulate monocyte proliferation. (A,B) Human precursors were cultured with MCSF (33 ng/ml) and RANKL (33 ng/ml) and the medium was changed on day 1 (MC d1), day 3 (MC d3) or day 5 (MC d5) to either medium containing MCSF only and no RANKL (A) or medium containing both MCSF and RANKL (B). Negative control (NC) are cells cultured with MCSF only; positive control (PC) are cells cultured with MCSF and RANKL, NC and PC were performed without media change. The samples were fixed on days 4–8 and the numbers of NFATc1-positive monocytes, the percentage of NFATc1-positive monocytes, numbers of monocyte and osteoclast nuclei were quantified using high content imaging. Data are means ± SEM, *n* = 6 replicates from 1 of 4 independent experiments. (C) Schematics of the role of RANKL-induced autocrine factors in monocyte proliferation and differentiation into osteoclasts.

To examine if continuous presence of RANKL is required for activation of NFATc1, we performed the experiments in which on different days of culture, the original MCSF and RANKL-containing medium was replaced by the fresh medium containing MCSF, either without RANKL (Fig. 5A) or with freshly added RANKL (Fig. 5B). It is important to note that no media change is recommended for human osteoclast differentiation assay, therefore the negative and positive control cultures were maintained in the absence or presence of RANKL, respectively, without media change. We have found that removal of RANKL from the media resulted in a reduction in the numbers and percentage of NFATc1-positive monocytes (Fig. 5A*i*,*ii*), and attenuation in both monocyte and osteoclast numbers (Fig. 5A*iii*,*iv*). The effect of RANKL withdrawal was evident even if it was removed on day 5 of culture. These data indicate that RANKL is required for continuous stimulation of NFATc1 and induction of osteoclastogenesis, as well as proliferation of monocytic precursors. Interestingly, we have found that even when RANKL was reinstated in the fresh media, media change still resulted in attenuated osteoclastogenesis (Fig. 5B). We observed that even though the percentage of NFATc1-positive monocytes was maintained after the media change, the numbers of NFATc1-positive monocytes were reduced (Fig. 5B*i*), reflecting the reduced total numbers of monocytes (Fig. 5B*iii*). These data suggest that *a*) RANKL-induced active monocyte proliferation is important for optimal osteoclastogenesis (Fig. 5C), and *b*) this monocyte proliferation is regulated at least in part by RANKL-induced release of autocrine factors from differentiating precursors, which are depleted during the change of culture medium.

### Identification of autocrine factors produced during osteoclastogenesis

**Fig. 6.**
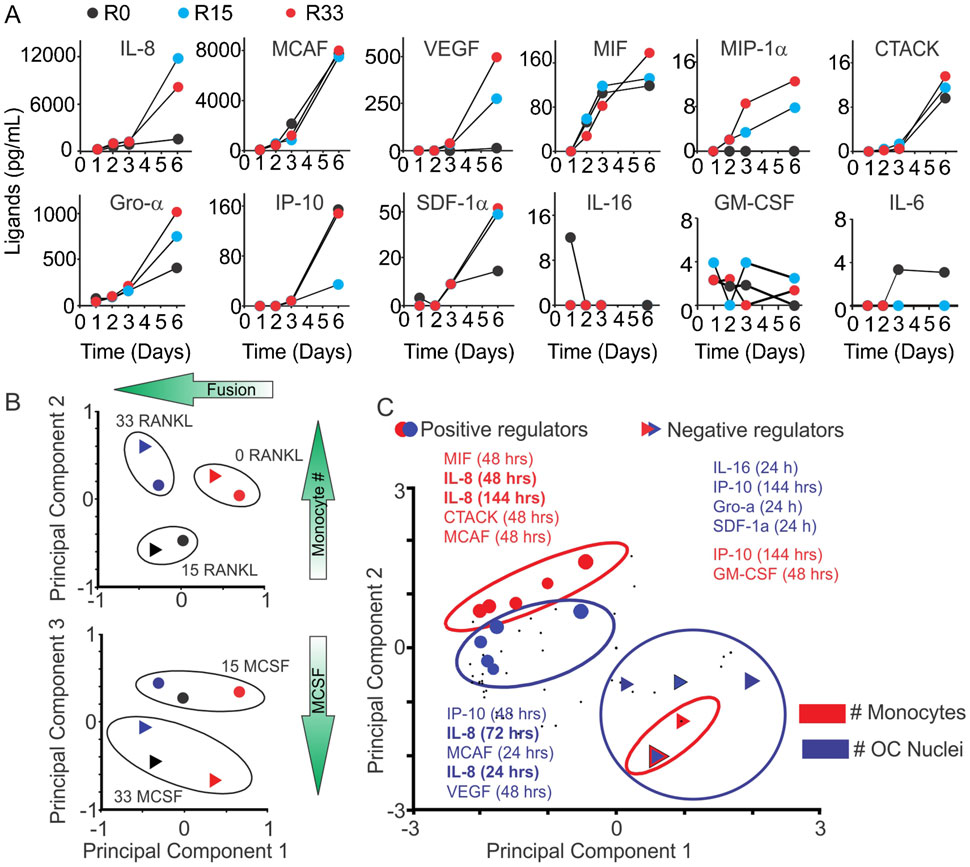
Cytokines produced during osteoclast differentiation. Human osteoclast precursors were cultured with MCSF (15 or 33 ng/mL) and RANKL (0, 15 or 33 ng/mL) for 24, 48, 72, 144 h and cytokine profiling in conditioned medium was performed using a 47-cytokine multiplex immuno-assay. (A) Time- and [RANKL]-dependence for the production of 12 cytokines during osteoclastogenesis. (B) Cytokine input scores validating that PLSR analysis clustered similar treatments. (C) Cytokine weights (input loadings) for principal components 1 and 2.

To examine if cytokine production is associated with osteoclast differentiation, we collected the conditioned medium throughout osteoclast differentiation and profiled cytokine levels with a bead-based multiplexed immunoassay. Twenty six of 47 cytokines were present at measurable levels. We examined the time and RANKL-concentration dependences for a representative set of cytokines (Fig. 6A). Some cytokines (such as MCAF, IP-10, MIF and CTACK) increased with time independent of RANKL treatment, some (such as IL-16 and IL-6) decreased with time, and some (such as IL-8, Gro-α, VEGF and MIF-1α) were increased in time- and [RANKL]-dependent manner (Fig. 6A). To interpret the potential roles of these cytokines during osteoclast formation, a Partial Least Squares Regression (PLSR) model was developed. The PLSR model identifies the relative importance of a set of predictors (cytokine measurements) that best correlates with the outcome response (osteoclastogenesis). The predictor dataset comprised the levels of 47 cytokines at 4 time points (24, 48, 72 and 144 h; 47×4 = 188 predictors) and the response dataset included the numbers of monocytes and osteoclasts observed on day 7 (2 responses). The inputs and responses were measured in six different conditions: 0, 15, or 33 ng/mL RANKL with either 15 or 33 ng/mL MCSF. To confirm that the model was identifying expected drivers of variability, the predictor scores were plotted for each of the 6 experimental conditions (Fig. 6B). Similar experimental conditions clustered together, with principal component 1 accounting for over 75% of the variance in the dataset. Since the dominant process occurring in these experiments is fusion, which is strongly dependent on RANKL, we labeled the corresponding *x*-axis on the Fig. 6B as “fusion”. Principal component 2 explained ∼15% of the variance in the dataset and corresponded to a shift upwards for the number of monocytes (*y*-axes on the Fig. 6B, top). The principal component 3, which separated the dose effect of MCSF, explained less than 8% of the dataset variance. To visualize the correlation of each of the 188 predictors (i.e. a specific cytokine level at a specific time point) with the osteoclastogenesis responses, the predictor values were plotted on the same axes of principal components 1 and 2 (Fig. 6C). The predictors that were shifted left on the *x*-axis acted as positive regulators of fusion and those that were shifted to the right were negative regulators of fusion. Shifts in the vertical direction select for positive (shifted upwards) and negative (shifted downwards) regulators of monocyte numbers. Two metrics from the PLSR model are commonly used to rank the relative importance of the predictors, the regression coefficients and the VIP score. Since the ranking of any single predictor has the potential to be generated by noise, we utilized the redundancy of measuring the same cytokines over four time points and used a sum of four scores to determine a cumulative score, which reflects both the strength of association of cytokine changes with outcome at the individual time point and a consistency of its predictive value across different time points (Tables 1, 2, 3, indicated as large symbols on Fig. 6C). Significant overlap between cytokines important for increase in monocyte and osteoclast nuclei was observed. In particular, interleukin 8 (IL-8, also known as CXCL8, a cytokine that acts through CXCR1 and CXCR2 receptors), vascular endothelial growth factor (VEGF) and monocyte chemotactic protein-1 (MCP-1) were found to be strongly associated with positive regulation of both monocytic and osteoclastic components of osteoclastogenesis (Tables 1, 3).

**Table 1.**
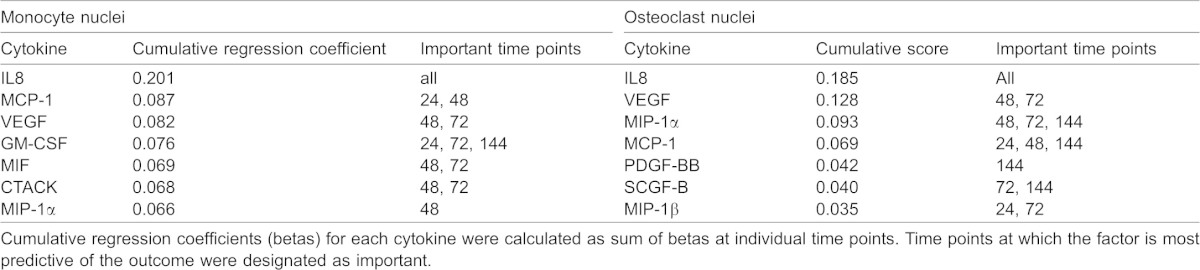
Top 7 cytokines identified as positive regulators during osteoclastogenesis

**Table 2.**
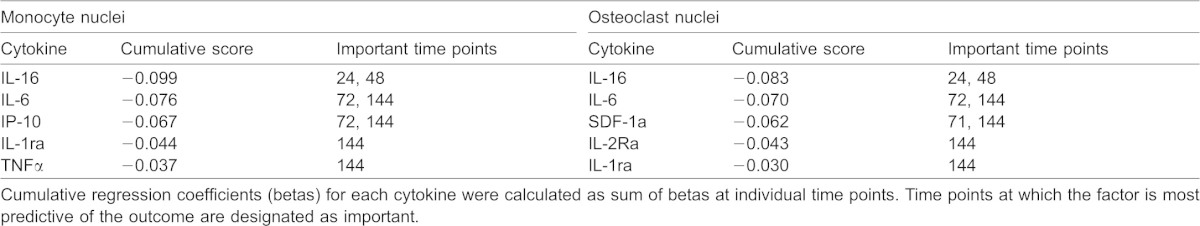
Top 5 cytokines identified as negative regulators during osteoclastogenesis

**Table 3.**
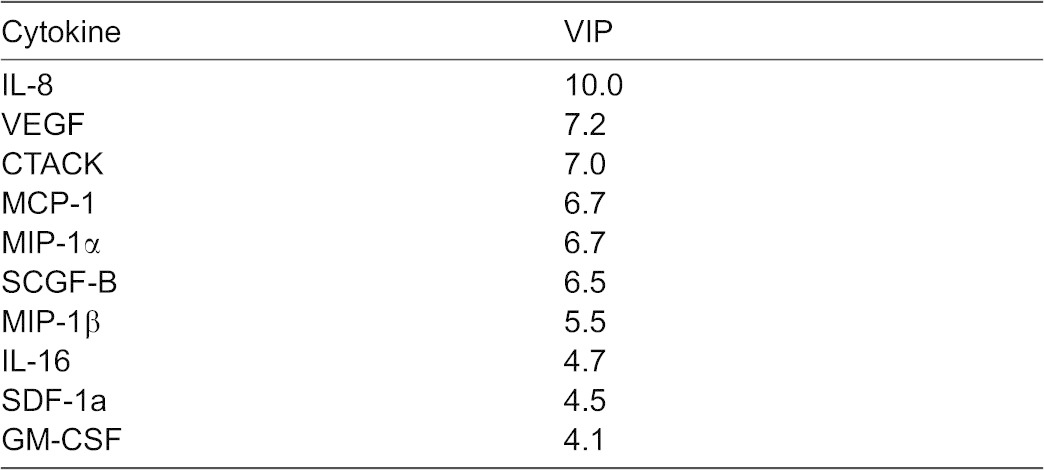
Cumulative very important parameter (VIP) scores for top 10 predictors

### Role of IL-8 in osteoclastogenesis

**Fig. 7.**
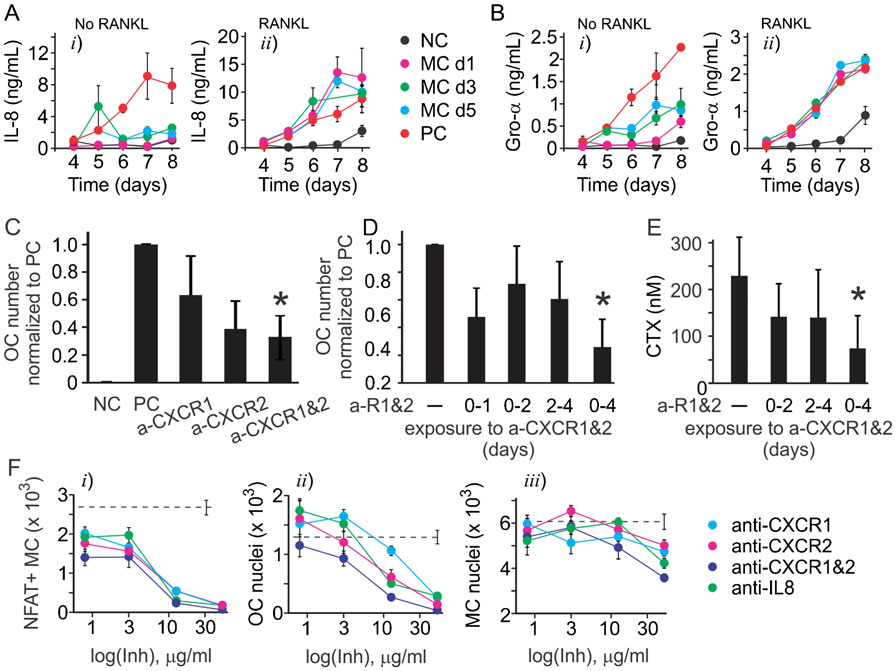
IL8 acts as autocrine factor during osteoclastogenesis. (A,B) Human precursors were cultured with MCSF (33 ng/ml) and RANKL (33 ng/ml) and the medium was changed on an indicated day (MC d1 for day 1 etc.) to medium containing MCSF only (no RANKL, *i*) or medium with MCSF and RANKL (RANKL, *ii*), and the production of IL8 (A) and Gro-α (B) was assessed. Data are means ± SEM, *n* = 3 replicates from 2 independent experiments. (C–F) Human precursors were treated with MCSF (33 ng/ml) and RANKL (33 ng/ml) in the absence (positive control, PC) or presence of antibodies blocking CXCR1 (a-CXCR1), CXCR2 (a-CXCR2), the combination of CXCR1 and CXCR2 blocking antibodies (a-R1&2), or anti-IL8 neutralizing antibody (50 µg/ml) for 1–7 days. Negative control (NC) are cells cultured with MCSF (33 ng/ml) only. (C) Average numbers of osteoclasts formed in indicated conditions. Data are means ± SEM, *n* = 2–5 independent experiments, *p<0.01 compared to positive control as assessed by t-test. (D,E) The combination of CXCR1 and CXCR2 inhibitors was most effective in inhibiting osteoclast formation (D) and resorption (E) when added for day 0–4. Data are means ± SEM, for panel D *n* = 3 independent experiments, for panel E *n* = 2 independent experiments, *p<0.05 compared to positive control as assessed by t-test. (F) Inhibitor concentration-dependences for their effect on NFATc1-positive monocytes, osteoclast nuclei and monocyte nuclei. Data are means ± SEM, *n* = 3 replicates from 1 of 2 independent experiments.

We next focused on validating the role of IL-8 in autocrine regulation of osteoclast formation, since its function in osteoclastogenesis has not been previously described. We examined if the media change during osteoclastogenesis affects the production of IL-8 (Fig. 7A) and GRO-α, which can act through similar receptors as IL-8, but was not identified and a predictor of osteoclast formation (Fig. 7B). When RANKL was removed during media change, the production of both IL-8 and GRo-α was attenuated, but IL-8 was affected to a higher degree (Fig. 7A*i*,B*i*). When fresh RANKL was added during media change, the production of both IL-8 and Gro-α was rescued, but while Gro-α was produced at the levels similar to positive control, IL-8 production was increased to higher than control values (Fig. 7A*ii*,B*ii*). Using the pharmacological inhibitors of CXCR1 and CXCR2, we have found that in the presence of individual inhibitors osteoclast numbers demonstrated the trend to decrease, which did not reach statistical significance. However, inhibition of both CXCR1 and CXCR2 resulted in significant attenuation of osteoclastogenesis (Fig. 7C). When added continuously for the first 4 days (but not day 0 to 2 or 2 to 4), the combination of CXCR1 and CXCR2 inhibitors resulted in inhibition of osteoclast formation (Fig. 7D) and resorption (Fig. 7E). Neutralizing IL-8 antibody also significantly and dose-dependently inhibited NFATc1 nuclear translocation (Fig. 7F*i*) and osteoclast formation (Fig. 7F*ii*), as well as less potently monocyte proliferation (Fig. 7F*iii*).

## DISCUSSION

Our data indicate that during osteoclastogenesis from human osteoclast precursors, several stages of differentiation can be identified. Initial stage (24–48 h) is characterized by active monocyte proliferation mediated in part by RANKL-stimulated release of autocrine factors. In the intermediate stage (48–96 h), osteoclast precursors develop calcium oscillations which lead to persistent nuclear translocation of NFATc1. In the final stage (120 h–9 day) precursors fuse and grow, resulting in appearance of large multinucleated resorption-capable osteoclasts. IL-8 (a cytokine important for the function of human immune system, which has no single homologue in mouse) was identified as a key regulatory autocrine factor produced downstream of RANKL during osteoclastogenesis. We have found that inhibition of IL-8 signaling interfered with osteoclastogenesis, and that in the absence of IL-8 activation of NFATc1 was inhibited. This study demonstrates how the regulation of differentiation can be understood using a primary human progenitor cell model with traditional techniques and novel high content imaging, proteomic and computational methods.

The power of *in vitro* progenitor cell systems to enable studies of pathologic mechanism and accelerate therapeutic design is beginning to be realized. Understanding of central nervous system development (Okada et al., 2008), molecular aspects diabetic cardiomytopathy (Song et al., 2011), and cardiotoxicity profiles of cancer therapeutics (Reynolds et al., 2012) have been made possible by recapitulating complex biology via progenitor cell culture. Using primary human osteoclast progenitor cells, we have characterized novel regulators of osteoclastogenesis generating understanding of both normal physiology and potential novel therapeutic targets. These insights were generated using high throughput measurement of single cell phenotype and signaling, multiplex cytokine profiling, and partial least square regression analysis to enable data-driven hypothesis generation from the resulting large, complex dataset.

The application of high content imaging to problems in pharmacology and toxicology counts several decades of successful history (Shariff et al., 2010); however, the use of this technique to understand cell differentiation is in its relative infancy. Several challenges need to be addressed in order to develop an image analysis platform that has a potential for universal application to this type of studies. First, it is essential to track the appearance of differentiated cells in the population of their precursors. While expression of certain gene or protein marker that can be fluorescently labeled represents a conventional readout, it does not by itself signify successful cell differentiation. As we demonstrate in this study, time- and [RANKL]-dependences for the expression of well characterized osteoclast marker genes (Boyle et al., 2003) were strikingly different from time- and [RANKL]-dependences for formation of morphologically and functionally identifiable osteoclasts. Therefore, it is imperative to automate identification of morphological cell attributes. An important feature of osteoclasts, which is also relevant to myocyte differentiation, is their multinucleation due to fusion of precursors. Large cytosol expansion resulting in considerable spacing between individual nuclei of a single osteoclast further complicates automatic cell identification. In the recent studies of myocyte differentiation, commercial MetaXpress application modules for cell sorting and angiogenesis tube formation were customized to track the formation of myotubes in culture (Arya et al., 2013; Scott et al., 2013). However, similar adaptations are not practical for identifying osteoclasts, which do not exhibit rigid form, and are very heterogeneous in their appearance, ranging from 3 to 20 nuclei per cell, which can be 20–500 µm in diameter. In this study, we developed additional algorithms for a recently developed high content microscopy image analysis program ImageRail (Millard et al., 2011), which allows automatic identification of subpopulations of osteoclasts and monocytes. Using this approach we were able to increase at least tenfold the number of conditions carried per experiment, and to obtain detailed information regarding behavior of these two cell populations in differentiating cultures. The new detailed information regarding osteoclast differentiation allowed us to roughly divide osteoclastogenesis in 3 phases: 1) early phase – from induction to day 3, characterized by minimal morphological changes, significant monocyte proliferation and induction of osteoclast markers TRAP, CtsK and MMP9, 2) intermediate phase – day 3–4, characterized by changes in calcium signaling and NFATc1 nuclear translocation ultimately resulting in synchronized cell fusion and 3) late phase – day 5–9, characterized by extensive fusion, cell growth, and continued induction of genes important for osteoclast function, MMP-9 and CtsK. In addition, the media change experiments demonstrated that even if RANKL was reinstated after the early media change, osteoclastogenesis was still attenuated, thus implying that autocrine factors induced by RANKL are critical for successful osteoclastogenesis from human precursors. Interestingly, similar media change experiments performed with mouse precursors do not result in attenuation of osteoclastogenesis (Boraschi-Diaz and Komarova, 2014), strongly suggesting that this aspect of regulation of human osteoclastogenesis cannot be studied using mouse models.

To identify autocrine mediators of osteoclastogenesis, we examined the time-, [RANKL]- and [MCSF]-dependence for the release of 47 different cytokines during the early and intermediate stages of osteoclast formation. We have found that 26 of 47 cytokines were present at measurable levels at least at one time point during osteoclast differentiation. Different patterns of changes of these cytokines with time and with the variation in RANKL and MCSF concentrations were observed; however, the intuitive analysis of the behavior of 26 cytokines at 4 time points at 6 different conditions is challenging. To reduce the complexity of the system and identify the most plausible candidate cytokines involved in regulation of osteoclastogenesis, we developed a PLSR model, which aims to extract the linear combinations of the predictors (factors in the signal dataset), which optimally explains the variation in response (osteoclastogenesis outcomes). The model predicted significant involvement of several cytokines in the regulation of osteoclast formation both in a positive and negative manner. Importantly, the cytokines demonstrated to regulate monocyte and osteoclast numbers exhibited significant overlap, further confirming that initial monocyte proliferation is important for successful osteoclastogenesis. Three cytokines were identified by the model as positive regulators of osteoclast and monocyte nuclei: IL-8, MCP-1 and VEGF. Of these cytokines, MCP-1 (Miyamoto et al., 2009; Morrison et al., 2014) and VEGF (Trebec-Reynolds et al., 2010; Zhang et al., 2008) were previously implicated as autocrine regulators of osteoclast formation and function consistent with model predictions. In contrast to previous reports demonstrating that TNFα plays a positive role as an autocrine regulator of osteoclast differentiation from mouse precursors (Nakao et al., 2007; Zou et al., 2001), we did not observe strong correlation of this factor with formation of human osteoclasts. Importantly, cytokine screening in combination with PLSR modeling allowed us to identify a novel autocrine regulator of osteoclast formation – interleukin 8.

IL-8 (CXCL8) is a CXC chemokine known for its importance in the regulation of the acute inflammatory response (Remick, 2005). In addition, an increase in IL-8 levels was demonstrated at several diseases associated with osteoclast activation, including rheumatoid and psoriatic arthritis (Cho et al., 2007; Fitzgerald and Chandran, 2012), and several cancers exhibiting high propensity to metastasize to bone, such as breast (Bendre et al., 2005; Bendre et al., 2003), lung (Hsu et al., 2010), renal (Perut et al., 2009) and oral squamous cell carcinoma (Hwang et al., 2012). While it was shown more than 15 years ago that human osteoclasts produce unexpectedly high levels of IL-8 (Rothe et al., 1998), the functional significance of this phenomenon remained unknown. One of the potential explanations of the discrepancy between the demonstrated involvement of IL-8 in human diseases and lack of mechanistic insights into its action is the lack of IL-8 equivalent in rodents, which are most widely used to model human diseases. In this study, using human osteoclast precursors we demonstrated that IL-8 plays an important role as a RANKL-induced autocrine mediator of osteoclast formation. We have found that inhibition of IL-8 cognate receptors CXCR1 and CXCR2 or removing IL-8 using neutralizing antibodies significantly and dose-dependently attenuated osteoclastogenesis even in continuous presence of RANKL. The interference with NFATc1 nuclear translocation was identified as a mechanism mediating the inhibitory action of IL-8. The ability to affect NFAT activation was not previously attributed to CXCR1 or CXCR2 or other CXC receptors, with the exception of a single study that demonstrated that CXCR2 overexpressed in the Chinese hamster cells can induce NFATc1 translocation in response to treatment with exogenous IL-8 or Gro-α (Wolff et al., 2010). Thus, our data suggest a novel paradigm of IL-8 function during osteoclastogenesis from human osteoclast precursors.

Taken together, our data highlight important differences in osteoclast differentiation in humans and rodents, and identify an important role of positive autocrine feedback generated by RANKL-induced cytokines, including MCP-1, VEGF and IL-8, in regulating osteoclast formation. In addition to providing new insights into the regulation of osteoclastogenesis and signaling events involved in this process, we identified cytokines, in particularly IL-8, as potential therapeutic target for inhibition of osteoclast formation and function in rheumatoid and in neoplastic disorders, with a possible novel mechanism from existing RANKL inhibition therapies.

## MATERIALS AND METHODS

### Osteoclastogenesis

Human primary osteoclast precursors (OCPs, Lonza Inc., 2T-110) are derived from CD34+ progenitors by Lonza using a proprietary differentiation process. Osteoclast precursors were plated at 5,000 cells/cm^2^ and cultured in osteoclast growth basal media including supplements and growth factors (Lonza Inc., PT-8001). Medium was not replenished. On day 3 and 6, cells were fixed with 4% paraformaldehyde (10 min), washed with PBS and stained for tartrate-resistant acid phosphatase (TRAP) (Sigma, 387A-KT). Osteoclasts were identified as multinucleated (more than 3 nuclei) TRAP-positive cells. For resorption studies, human osteoclast precursor cells were plated on an OsteoAssay human bone plate (Lonza Inc., PA-1000). After indicated time, conditioned medium was collected and collagen degradation products (CTX) were measured using CrossLaps for culture ELISA (ids AC-07F1).

### Manual osteoclast quantification

Osteoclasts were identified as multinucleated (more than 3 nuclei) TRAP-positive cells and were further characterized by image analysis. Images were recorded using a digital camera linked to PixeLINK Capture SE® software (PixeLINK, Ottawa) at 40× magnification for cell surface analysis and 100× magnification for evaluation of number of cell nuclei. For each experimental condition, the cell surface area and nuclei number of each multinucleated TRAP positive osteoclastic cells were evaluated.

### High content imaging and automated osteoclast quantification

OCPs were cultured on 96-well ViewPlates (PerkinElmer), fixed in 4% paraformaldehyde for 10 min, and stored in PBS at 4°C. Cells were permeabilized with 0.1% Triton X-100 for 5 min, blocked with Odyssey blocking buffer (Li-Cor Biosciences) and incubated with primary antibodies (NFATc1 (H-110), Santa Cruz, sc-13033; PE Mouse Anti-Human CD51/CD61, BD Biosciences, 550037) overnight at 4°C with agitation. Cells were washed in PBS with 0.1% Tween 20 (PBS-T) and incubated with donkey anti-rabbit Ab IgG AlexaFluor® 647 (Life Technologies, A31573) or for 1 hr at room temperature with agitation. After washing again with PBS-T, cells were incubated with Hoechst 33342 (Life Technologies, H3570), Whole Cell Blue (Thermo Scientific, 8403501), Alexa Fluor® 660 phalloidin (Life Technologies no. A22285) for 30 min at room temperature. Images were acquired with an ArrayWorx Multi-Format Reader (Applied Precision, Issaquah, WA).

Cell segmentation was performed using a custom osteoclast quantification module written for the open source software package, ImageRail (Millard et al., 2011). Briefly, the cytoplasm of osteoclasts was identified as regions with CD51/CD61 staining intensity above a user defined threshold. Nuclei were identified as regions above a user defined threshold for Hoechst 33342 intensity and nuclei within osteoclast cytoplasm regions were counted. Finally, the precursor cell cytoplasm was then identified around each nucleus that was negative for CD51/CD61 staining by user defined threshold for Whole Cell Blue intensity. ImageRail exported the number of osteoclast nuclei, number of precursors, and the mean NFATc1 pixel intensity for each precursor nucleus in each well. Precursor cells were gated into NFAT− and NFAT+ populations by fitting a bimodal Gaussian distribution to the NFAT histogram and setting the gate as the minimum between the 2 modes.

### RNA isolation

RNA was extracted and isolated using the RNeasy mini kit (Qiagen, 74104). Real-time PCR was performed using Applied Biosystems TaqMan gene expression assays for RANK (Hs00187192_m1) TRAP (Hs00356261_m1), Cathepsin K (Hs00166156_m1), MMP-9 (Hs00234579_m1), CTR (Hs01016882_m1) and beta actin (Hs99999903_m1).

### Microspectrofluorimetry

Human primary osteoclast precursors were plated on fibronectin (5 µg/ml) coated glass-bottom 35 mm dishes (MatTek Corporation) and cultured in basal media including RANKL (0, 15 or 33 ng/ml) and MCSF (15 and 33 ng/ml). Cells were incubated with basal media including ligands or not plus 10 mM HEPES and loaded at room temperature for 40 min with fura-2-AM (Invitrogen, F1221). Cells were washed twice and imaged using a fluorescence inverted microscope (Nikon T2000), cooled CCD Camera (Hamamatsu) connected to the image analysis software (Volocity, Improvision), which recorded fluorescence emission at 510 nm, following excitation at 340 and 380 nm alternated by a high-speed wavelength switching device (Lambda DG-4; Quorum Technologies). [Ca^2+^]_i_ was calculated using fura-2-AM calcium imaging calibration kit (Invitrogen, F-6774). Test compounds were added to the bath. Changes in [Ca^2+^]_i_ were measured at baseline for 120 sec.

### Immunoassays for quantifying cytokine abundance

Conditioned medium from OCP culture was stored at −20°C until analysis. Bioplex Pro Human Cytokine kits (Bio-Rad) were used to quantify cytokine levels according to the manufacturer's instructions. The following analytes were measured: IL-1β, IL-1ra, IL-2, IL-4, IL-5, IL-6, IL-7, IL-8, IL-9, IL-10, IL-12 (p70), IL-13, IL-15, IL-17, eotaxin, basic FGF, G-CSF, GMCSF, IFN-γ, IP-10, MCP-1 (MCAF), MIP-1alpha, MIP-1beta, PDGFBB, RANTES, TNF-α, VEGF, IL-1α, IL-2Ra, IL-3, IL-12(p40), IL-16, IL-18, CTACK, GRO-α, HGF, IFN-α2, LIF, MCP-3, M-CSF, MIF, MIG, β-NGF, SCF, SCGF-β, SDF-1α, TNF-β, TRAIL.

### High-throughput data modeling using Partial Least Squares

PLSR model fitting was adapted from previous work (Kumar et al., 2007) as follows. The predictor input matrix was 6 rows×188 columns, where the 6 rows were experimental treatments (0, 15, or 33 ng/mL RANKL with either 15 or 33 ng/mL MCSF) and the 188 columns consisted of the 47 cytokines×4 time points (1, 2, 3, and 6 days). Column-wise z-scoring was performed on the predictor matrix. Osteoclastogenesis readouts (number of monocyte and osteoclast nuclei) were quantified at day seven to obtain the corresponding output response data. We used the SIMPLS algorithm using the Matlab routine plsregress and the NIPALS algorithm, both of which yielded equivalent results. The importance of each input cytokine (at a particular time point) was assessed both by computing the VIP score, which accumulates the input importance over all outputs, and by the resulting regression coefficients, which quantify the importance of an input for each output separately.

### Inhibitor studies

Anti-CXCR1 (MAB330, R&D Systems), anti-CXCR2 (MAB331-100, R&D Systems), and anti-IL-8 (AB-208-NA, R&D Systems) blocking antibodies were diluted to 50 µg/ml in PBS and added to osteoclast cultures daily.

### Statistical analysis

Data are presented as representative images, representative traces or means ± standard error of the mean (SEM) with sample size (n) indicating the number of independent experiments. Differences were assessed by ANOVA or Student's t-test and accepted as statistically significant at p<0.05.
